# Preparation of Rod- and Urchin-Like Selenium Nanostructures by Hot-Injection and Application to Simple Flexible Coated Photosensors

**DOI:** 10.1021/acsomega.5c07956

**Published:** 2025-12-08

**Authors:** Kapil Patidar, Pen-Ru Chen, Hsueh-Shih Chen

**Affiliations:** † 34881 National Tsing Hua University, Department of Materials Science and Engineering, No. 101, Sec. 2, Kuan-Fu Road, Hsinchu 30013, Taiwan; ‡ College of Semiconductor Research, National Tsing Hua University, No. 101, Sec. 2, Kuan-Fu Road, Hsinchu 30013, Taiwan; § Department of Chemical Engineering & Materials Science, College of Engineering, Yuan Ze University, Taoyuan 32003, Taiwan

## Abstract

This study presents a synthetic approach for obtaining highly crystalline selenium (Se) nanorods (NRs) and urchin-like nanostructures. By modulating the postinjection cooling rate, the morphology of Se nanostructures can be precisely tuned, promoting anisotropic growth in the presence of supersaturated Se monomers. This method enables a rapid phase transformation from amorphous to trigonal Se under ambient pressure and low-temperature conditions. Furthermore, a simple, flexible light detector was fabricated via dip-coating Se nanorods onto PET, yielding an ON/OFF ratio of ≈10 and stable operation after more than 3000 bending cycles. This work demonstrates a rapid, scalable route for morphology-controlled Se nanostructures compatible with low-cost and flexible optoelectronic applications. The proposed method offers a promising strategy for producing high-quality one-dimensional (1D) nanomaterials.

## Graphical abstract

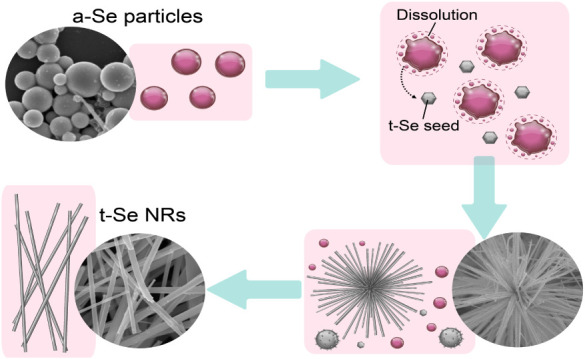

## Introduction

The physical and chemical properties of optoelectronic semiconductors make them highly versatile for a wide range of applications, including electronics, sensors, lighting, and catalytic devices.^1–10^ Recently, chalcogenide-based two-dimensional (2D) materials have garnered significant attention for their potential in photosensing applications due to their tunable band gaps and excellent light absorption properties.^11–13^ As an alternative, elemental selenium (Se) offers a simpler, cadmium-free fabrication route with intrinsic photosensitivity. Se is an essential trace element and a semiconductor with distinctive properties, including anisotropic thermal conductivity and high photoconductivity, which are attractive for flexible and low-cost optoelectronics.^14–16^

Crystalline Se can be obtained by slowly cooling saturated solutions of amorphous Se in hot aniline, where Se undergoes an amorphous-to-trigonal phase transition, yielding diverse nanostructures.^17^ Because morphology strongly influences the optoelectronic and mechanical responses, controlling this transformation is essential. Se nanostructures have been explored as building blocks for photodetectors, wearable piezoelectric devices, and lithium–selenium (Li–Se) batteries.^18–20^ However, slow-cooling routes often require extended times and solvent-specific conditions, motivating the development of rapid, ambient-pressure, and scalable synthesis with morphology control.

Several approaches have been developed to synthesize Se nanostructures, including laser ablation of Se powders,^21^ electrodeposition from an ionic liquid solution at high temperatures,^22^ and vapor growth methods that involve the thermal evaporation of Se powder with Si powder as a catalyst.^23^ Although these methods can produce high-quality Se nanostructures, they often require specialized equipment, high energy consumption, or complex processing conditions, limiting their practicality for large-scale production. Wet-chemical approaches commonly employ selenious acid/hydrazine precursors in hydrothermal,^24,25^ sonochemical,^26^ or liquid–liquid interface methods (often assisted with poly­(vinylpyrrolidone) (PVP) to promote crystallization and shape). However, such synthesis typically runs for several hours to days to complete, which can be a significant drawback when rapid synthesis is needed. Alternative synthesis techniques, such as those utilizing NaBH_4_ as a reducing agent or silver-assisted growth of Se nanowires (NWs) and nanorods (NRs), have also been reported.^27,28^ Despite these advancements, the development of a fast, controllable, ambient-pressure-scalable solution route for Se nanostructures remains desirable.

Building on related hot-injection chemistry, prior work reported the one-pot synthesis of ZnO NWs and Se NRs under different conditions.^29^ Using that approach, ZnO nanowires can be synthesized independently without the formation of Se products.^30^ Here, we combine hot injection with controlled cooling to produce phase-pure trigonal Se nanostructures with tunable morphology (nanorods vs urchin-like microstructures) at ambient pressure within tens of minutes using common solvents and glassware. We further correlate the solvent coordination and cooling protocol with observed anisotropic growth, and we demonstrate a flexible photodetector fabricated by simple dip-coating, highlighting the practicality of this rapid, morphology-controlled synthesis. This work aims to provide insights into the development of Se-based functional nanomaterials for future flexible technological applications.

## Results and Discussion

The crystal structure of Se NRs synthesized at 320 °C was characterized by X-ray diffraction (XRD), as shown in Figure 1a. All diffraction peaks are indexed to the trigonal structure of Se (t-Se) with a (101) preferred orientation (JCPDS card No. 06-0362). The calculated lattice constants (*a* = 0.435 nm, *c* = 0.494 nm, *c*/*a* = 1.134) are in good agreement with the reported trigonal phase (*a* = 0.437 nm, *c* = 0.496 nm, *c*/*a* = 1.135) in the literature.^31^ A Scherrer analysis of the t-Se (101) reflection (Cu Kα, λ = 1.5406 Å; *K* = 0.9), using the peak full width at half maximum (FWHM) after instrumental broadening correction, gives an apparent coherent-domain size of *D* ≥ 80 nm, indicating that the rods are at least single-domain across their diameter. The morphology of the obtained Se NRs is examined by SEM and TEM images, as shown in Figure 1b and Figure 1c, respectively. From the SEM image, the Se NRs show an average width of ∼80 nm and an average length of ∼12 μm, with the longest length up to ∼20 μm, far exceeding the Se exciton Bohr radius; thus, quantum confinement is negligible, and the gap is expected to be near bulk t-Se (∼1.8–2.0 eV). The length distribution of the obtained Se NRs is plotted in the inset of Figure 1b. The XRD-derived domain size is consistent with the ∼80 nm average width observed by SEM. In addition, the TEM images reveal a rod morphology of the sample consistent with that observed from the SEM images.

**Figure 1:**
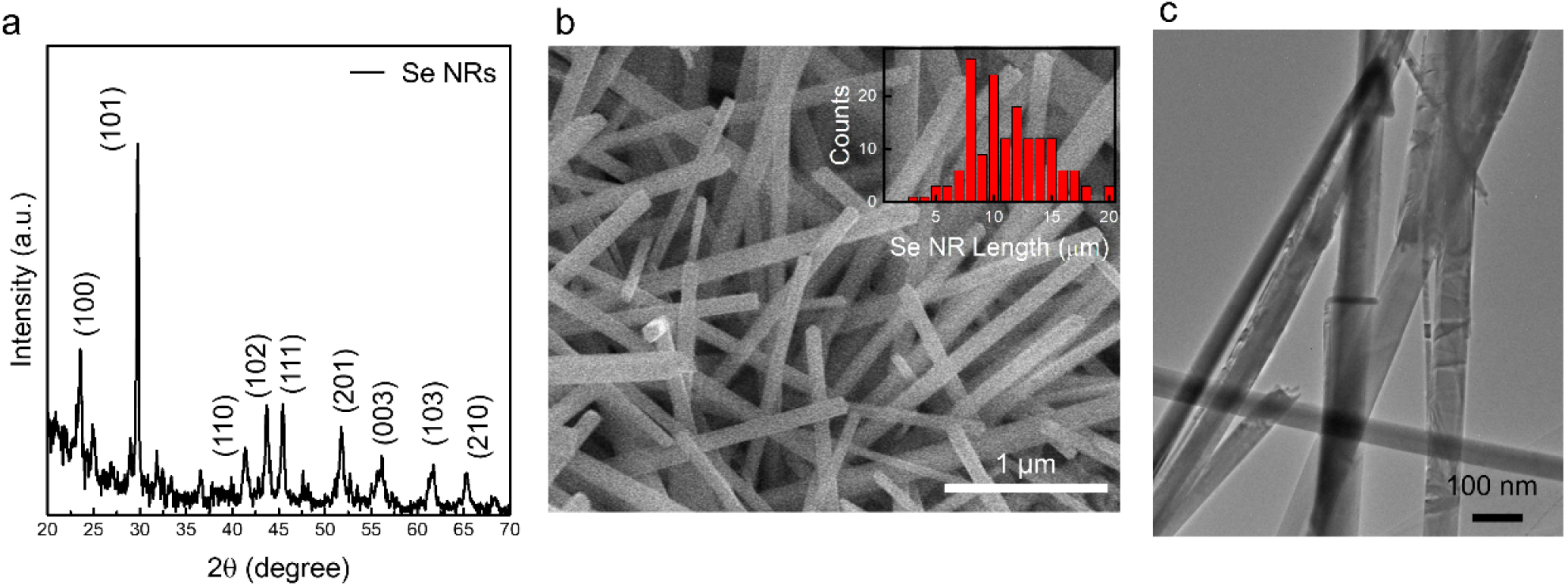
Characterization of Se NRs prepared at 320 °C. (a) XRD pattern. (b) SEM images. (c) TEM image. The inset in (b) shows the length distribution of Se NRs estimated from more than 200 rods. Note that the length might be underestimated because of tilting and breaks.

### The Roles of Growth Media (Solvents)

In previous hydrothermal approaches, Na_2_SeO_3_, H_2_SeO_3_, and hydrazine (N_2_H_4_) were employed for the growth, which generally required extended reaction times until equilibrium conditions.^17,32^ The present synthetic approach is based on the hot-injection quenching process; supersaturation leads to rapid nucleation of Se species within seconds after injection, while subsequent anisotropic crystal growth into nanorods occurs over the following tens of minutes. It has been found that both IPA and TOPO are essential for the formation of 1D nanostructures. As shown in Figure 2a, the Se product prepared in a mixture of IPA and TOPO exhibits long nanorod shapes. Interestingly, when IPA is used as the sole reaction solvent, the precipitates consist mainly of irregular particles with a diameter of approximately 500 nm and a small proportion of rod-like structures with a length of approximately 2 μm. In contrast, when only TOPO is utilized as the reaction solvent, a solution with a transparent light-yellow color is obtained without any precipitates (Figure S1). These results suggest that TOPO can dissolve Se powders, while IPA induces the precipitation of Se nanostructures. Furthermore, we replaced TOPO with a noncoordinating solvent, octadecene (ODE), to form a reaction solvent of IPA and ODE, while keeping other parameters constant. The resulting precipitates exhibited plate-like structures on the microscale, as seen in Figure 2c. The larger scale of the microstructure precipitates prepared from IPA and ODE may be due to the faster precipitation rate with the noncoordinating ODE compared to the relatively strong coordinating TOPO. Our results indicate that IPA is essential in synthesizing Se NRs, as it has been reported to facilitate polymer growth, which may also facilitate the growth of Se NRs.^33,34^ This IPA-assisted synthetic method offers potential for large-scale synthesis due to its simple process and short reaction time.

**Figure 2:**
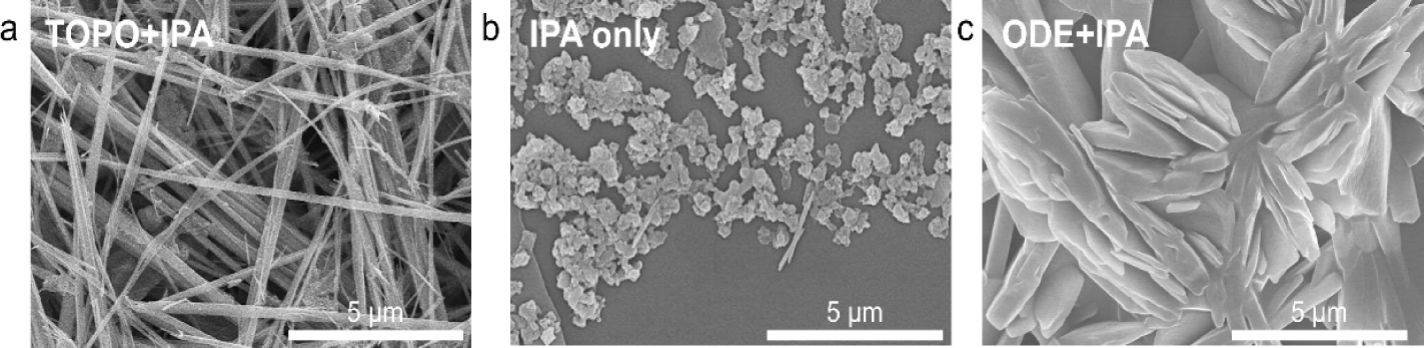
Se NRs prepared from a mixture of IPA and TOPO (a), pure IPA (b), and a mixture of IPA and ODE (c).

### Effect of Cooling Rate on the Growth

The melting point of Se is much lower (217 °C) than the reaction temperature (320 °C) used in the synthetic process. As a result, Se NRs form at a certain point during the cooling process, implying that the cooling rate is a crucial factor in determining Se growth. In the typical synthetic process (cooling rate ∼30 °C/min), the products are NRs (Figure 3a). For a process with a rapid cooling rate (∼300 °C/min), none of the NRs are produced. Instead, the resulting product consists of spherical particles, as shown in Figure 3b. The composition of the samples is mainly Se (85–90% Se, 10–15% C and P), as determined by SEM-EDS. The formation of smaller particles indicates that the growth of Se NRs requires more time. In addition, the observed phenomenon highlights the significance of controlling the cooling rate to achieve the desired morphology.

**Figure 3:**
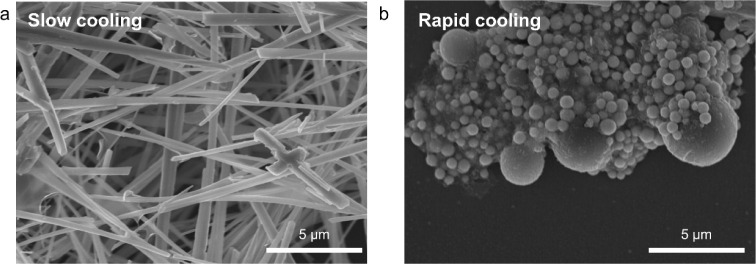
Se nanostructures prepared in the IPA/TOPO mixture with a relatively slow cooling rate (∼30 °C/min, naturally cooled) (a) and a rapid cooling rate (>300 °C/min, quenched in ethanol) (b).

### Growth Mechanism of NRs

The evolution of Se NR growth is investigated further at various time intervals during the cooling process (∼30 °C/min), as depicted in the SEM images presented in Figure 4. At 3 min, spherical particles are first produced (Figure 4a). According to EDS analysis, the composition of the samples is mainly Se (85–90% Se and 10–15% C and P). Some NRs are observed to be attached to the particles, indicating that nanorods form on the surface of the particles. At 5 min, the product consists of a mixture of spherical particles and long nanorods (Figure 4b). The images suggest that nanorods grow from the particles. At 10 min, the Se NRs become the primary phase (Figure 4c). Furthermore, some sea urchin-like microstructures composed of Se NRs are identified in the sample cooled for 10 min (Figure 4d). The results suggest that Se NRs transform from the particles.

**Figure 4:**
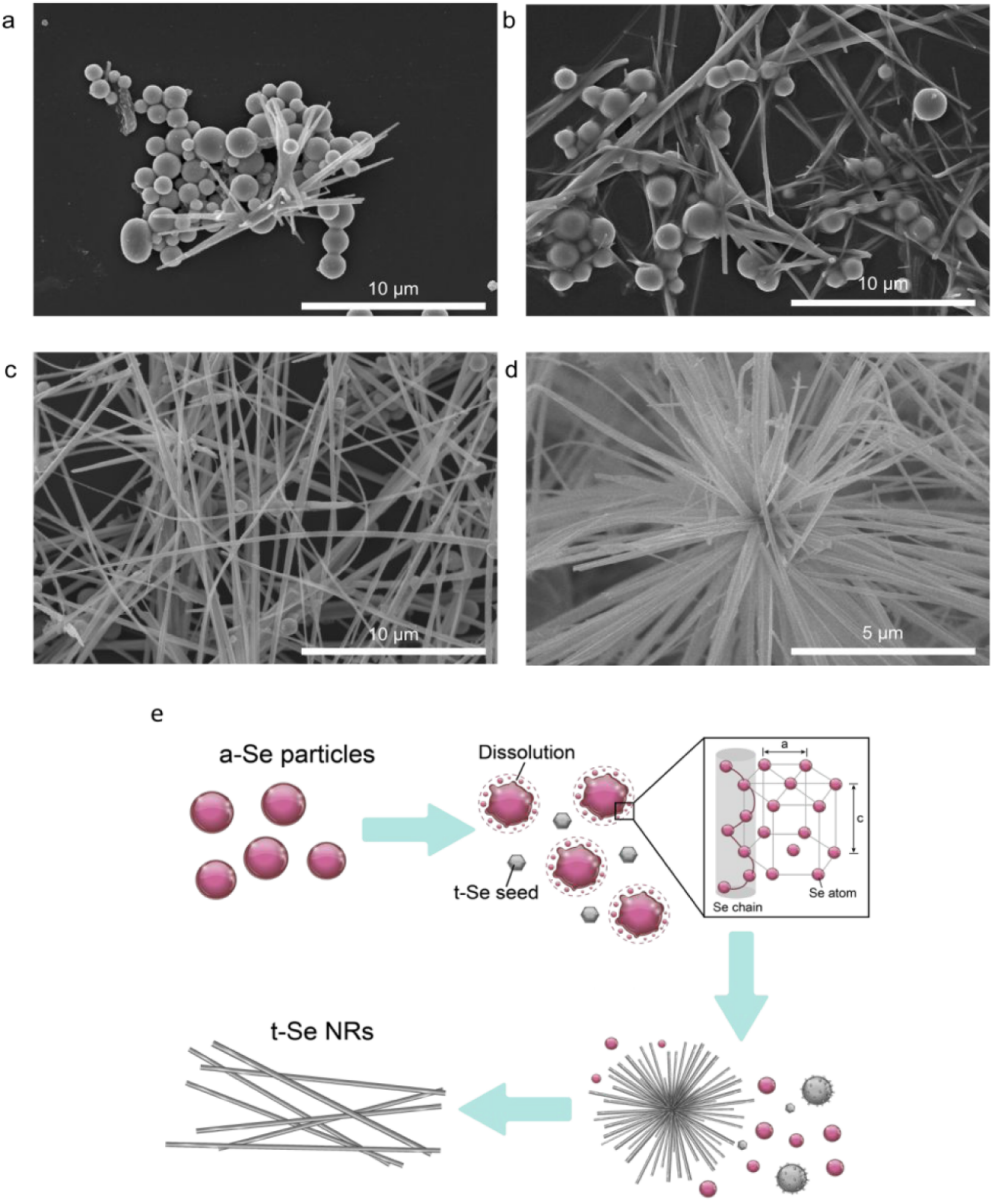
SEM images of Se nanostructures were collected at various time intervals: (a) 3 min, (b) 5 min, and (c) 10 min. (d) Sea urchin-like Se microstructures were found in the 10 min sample. The cooling rate is ∼30 °C/min. (e) Possible growth mechanism of Se NRs and sea urchin-like microstructures.

According to the developing morphologies observed from SEM, the growth mechanism of Se NRs in the hot-injection process is proposed to be similar to those from hydrothermal or solvothermal processes, in which amorphous Se (a-Se) spherical particles first form and transform into crystalline t-Se, followed by the formation of Se NRs.^12,17,23^ It has also been suggested that the crystal formation of 1D NR growth is directed by the helical chain of t-Se structures, as shown in the HR-TEM image of Se NR (Figure S2). In the current synthetic process, a-Se particles are the only product (brick-red color) in the early stage of the cooling process (∼3 min), while the crystalline Se NRs become the primary product (dark brown color) later in the cooling process (∼10 min) (Figure S3–S4).^9^ It has been known that a-Se particles are metastable and easily dissolve or transform into crystalline t-Se,^35^ acting as seeds for growing Se NRs.^12,36^ Therefore, a proposed growth mechanism of Se NRs in the hot-injection cooling process is schematically illustrated in Figure 4e. The oversaturated Se monomers precipitate and deposit on the Se seed particles, forming crystalline Se NRs during the cooling process. Thus, the growth of Se NRs on the Se seeds can lead to a microstructure like that of a sea urchin, as shown above.

### Preparation of Te and S NRs

A similar synthetic process has also been employed to prepare Te and S nanorods (NRs), as shown in the TEM images in Figure 5a–b. The growth mechanism of Te NRs is expected to be similar to that of Se NRs, as a similar growth phenomenon is observed. Representative SEM images and XRD patterns are provided in Figures S5–S6. Without additives, Te NRs exhibit diameters of ∼5 μm and lengths of 60–200 μm; introducing a ZnO additive markedly reduces the rod diameter to 0.6–2.5 μm and the length to 9–16 μm without altering the crystalline phase, as indicated by essentially identical XRD peak positions and profiles (Figure S6a). We therefore ascribe the size reduction to additive-mediated nucleation/growth regulation rather than a phase change. Furthermore, when exposed to an electron beam during TEM observation for several minutes, Te and S NRs transform into tubular structures (Figure 5c–d). HR-TEM further reveals that Te rods are frequently anchored to residual particulate domains (Figure S7), consistent with a seeded growth-dissolution-recrystallization pathway and occasional oriented attachment during anisotropic elongation. TEM-EDS on individual rods (Figure S8) confirmed Te as the dominant elemental signal. Additionally, preparation of Te and S NRs has also been reported previously.^37,38^ Moreover, unlike previously reported transformations of Se nanotubes into Se NRs,^39^ the structural transformation observed in this study occurs in the opposite direction, from NRs into nanotubes. The underlying mechanism of this transformation remains unclear and is currently under investigation.

**Figure 5:**
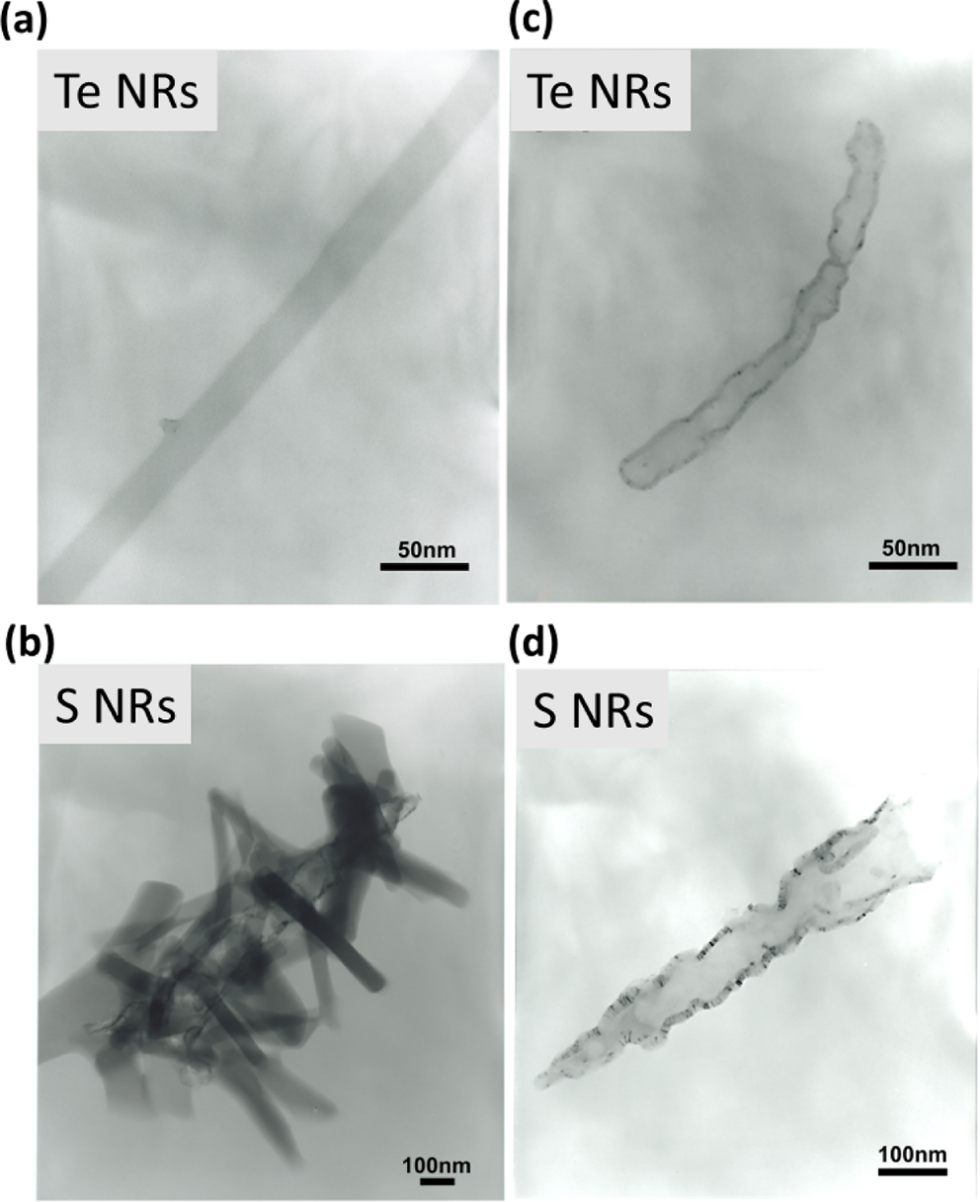
TEM images of Te NRs. (a) Selection of a Te NR. (b) A Te nanotube transforms from the NR in image (a) after exposure to the electron beam. EDS and SEM images of Te NRs are provided in the Supporting Information. TEM images of S NRs (c) and a tubular S structure (d). See Figures S5 and S6 for SEM and XRD of Te and S NRs.

### Application to Coated Photosensors

To demonstrate the potential applications of Se NRs synthesized by the hot-injection-based cooling process, a simple method is used to fabricate a photosensor by directly dip-coating Se NRs onto a plastic poly (ethylene terephthalate) (PET) film, as shown in Figure 6c. The as-synthesized Se NRs, dispersed in toluene, are directly dip-coated onto the plastic film under ambient conditions. A commercial silver conductive paste is then applied to the coated Se NR film, enabling the instant generation of a photocurrent under room light. At 1 V bias, the device exhibited a dark current of ∼1 pA and a photocurrent of ∼10–12 pA under room lighting (∼250 cd/m^2^), corresponding to an on/off ratio of ∼10 (Figure 6d). To clarify the operating mode, I–V characteristics measured in the dark and under room light are provided in Figure 6a. The curves exhibit no measurable short-circuit photocurrent at 0 V, and illumination increases the I–V slope, establishing photoconductive, not photovoltaic, behavior. The absolute current is limited by Schottky-limited hole injection at Ag/Se, inter-rod junction resistances in the percolating film, single-pass coverage (coffee-ring inhomogeneity), and the low irradiance of ambient illumination rather than by intrarod crystallinity. Mechanical deformation modulates the network transport. As shown in Figure 6b, bending the PET substrate to ∼60° increases the I–V slope (conductance), consistent with improved percolation/contact between adjacent rods on the curved surface.

**Figure 6:**
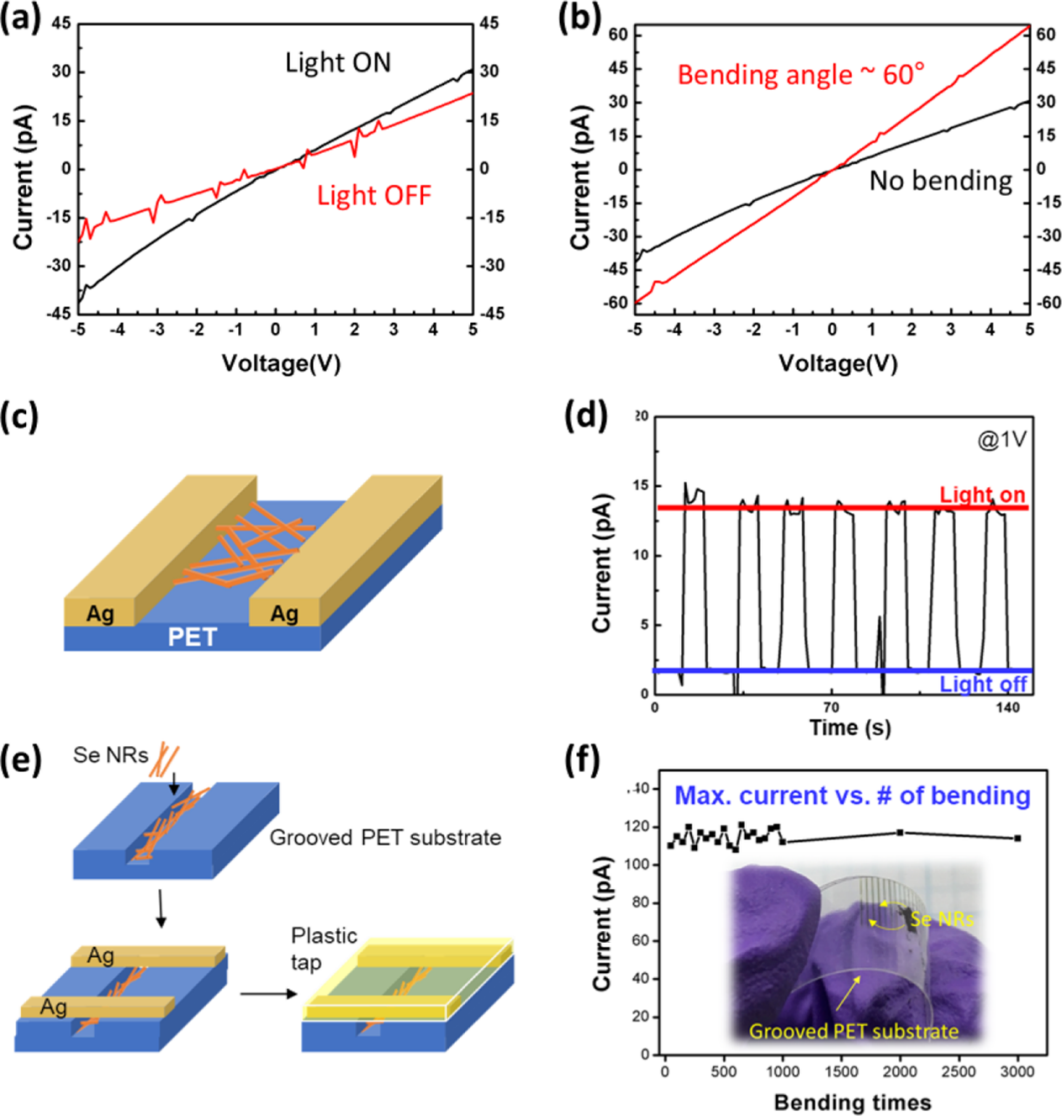
Fabrication of a simple light sensor with Se NRs. (a) I–V characteristics of the flat, dip-coated Ag/Se-NR/Ag device on PET under dark and room-light illumination. No measurable short-circuit photocurrent is observed at 0 V, consistent with photoconductive operation. (b) I–V under bending (∼60°) for the same flat device, showing increased slope (conductance) relative to the flat state. pA-level noise reflects instrument bandwidth near the current floor. Schematic of the flat device (c) and time-trace at 1 V under room-light switching (d). Grooved-PET device: (e) schematic of groove-guided casting and (f) maximum photocurrent at 1 V vs bending cycles, showing improved photocurrent (∼110 pA) and stability for >3000 cycles (inset image of the grooved PET with Se NRs). The grooved structure was prepared with a utility knife.

Although the absolute photocurrent lies in the picoampere range, the clear and stable switching behavior highlights the feasibility of this simple fabrication route. In the lateral Ag/Se-NR/Ag photoconductor, t-Se behaves as p-type; in the dark, Schottky barriers at Ag/Se and the wide bandgap yield a low dark current. Under room-light illumination, photogeneration and partial electron trapping at surface/junction states produce photogating, which lowers the effective hole-injection barrier and increases the conductivity of the percolating NR network, consistent with the observed ∼10 times ON/OFF ratio. A qualitative band diagram and extended discussion are provided in the SI (Note S1 and Figure S9). Since the photocurrent is generated through the conductive paths formed by overlapping Se NRs, the coffee ring effect can impact the distribution and dispersion of the NRs, thereby influencing the photocurrent. To further improve device performance, a modified photosensor structure was proposed for Se NRs, which were cast onto a grooved PET substrate, as shown in Figure 6e. Improved photocurrent can be observed when Se NRs are allocated in the grooves (∼110 pA at 1 V under room light), and the generated photocurrent remains reliable even after bending more than 3000 times (Figure 6f). This method presents a simple approach for utilizing Se NRs in optoelectronic applications due to its simplicity, cost-effectiveness, and ease of fabrication. Compared with conventional light sensors, Se NRs offer several advantages, including high sensitivity, a scalable production process, and compatibility with flexible substrates. Moreover, the successful demonstration of a Se NR-based photosensor highlights the feasibility of using Se NRs synthesized through a hot-injection-based cooling process in various optoelectronic devices. Our findings align with these studies, reinforcing the idea that solution-processed Se NRs can serve as effective components in optoelectronic devices. The ability to fabricate light sensors using a straightforward dip-coating process suggests that Se NRs could be adapted for large-area, low-cost production. Furthermore, the strong photocurrent response of the Se NR-based sensor under ambient light conditions (∼250 cd/m^2^) indicates that these nanorods could be incorporated into self-powered photodetection systems or hybrid energy-harvesting devices. Moreover, further investigations into the long-term stability, response speed, and spectral selectivity of Se NR-based photodetectors will be essential to advancing their practical applications.

## Conclusions

In summary, a hot-injection-based quenching method has been developed to synthesize highly crystalline Se nanorods. The morphology of the Se products, including rod-like and urchin-like structures, can be controlled by adjusting the cooling rate of the reaction solution. The growth mechanism has been investigated, revealing that amorphous Se (a-Se) particles first form and subsequently evolve into sea urchin-like microstructures and Se nanorods. Furthermore, a simple light sensor has been fabricated by directly dip-coating Se nanorods onto a plastic film with Ag paste under ambient conditions, demonstrating their potential for optoelectronic applications. These findings suggest that Se nanorods synthesized through this method could serve as promising materials for next-generation photodetectors and flexible sensing devices. The straightforward fabrication process, combined with the tunability of Se nanostructures, offers new opportunities for scalable production and integration into multifunctional electronic systems.

## Experimental Section

### Chemicals

Isophthalic acid (IPA, 99%), trioctylphosphine oxide (TOPO, 90%), selenium powder (Se, 99.99%), tellurium powder (Te, 99.99%) and sulfur powder (S, 99.98%) were purchased from Sigma-Aldrich. Tri-*n*-butylphosphine (TBP) was obtained from Kanto Chemical Co., Inc. All of the reactants were used without further purification.

### Synthesis of Se NRs and Sea Urchin-Like Se Microstructures

The synthesis of Se NRs and sea urchin-like Se microstructures was modified from hot-injection methods.^25,40,41^ Briefly, 1.5 mmol of Se powder was dissolved in 3 mmol of TBP by sonication to form a clear, transparent solution as a Se stock solution. Twenty mmol of IPA and 10 mmol of TOPO were added into a three-neck flask filled with Ar and heated to 150 °C for 10 min to remove undesired trace water. The mixture was then heated to 320 °C, and the Se stock solution was rapidly injected into the mixture. After injection at 320 °C and a 20-min reaction, the mixture was cooled either (i) naturally by switching off the heater and allowing the flask to cool under Ar (∼30 °C/min) or (ii) rapidly by discharging the hot mixture into excess room-temperature ethanol (∼300 °C/min). Cooling rates were obtained from recorded temperature–time profiles as average slopes. The obtained Se NRs were precipitated by adding a mixture of ethanol and toluene, followed by centrifugation at 5000 rpm for 3 min. The supernatant was discarded, and the centrifugation process was repeated three times. The precipitated Se NRs were redispersed in ethanol for subsequent analyses. Aliquots were collected and quenched in ethanol to study the cooling rate. Photodetector measurements under ambient light were performed under standard room lighting (∼250 cd/m^2^) without the use of any additional external light source.

### Synthesis of Te Microstructures and S Nanostructures

The Te microstructures were prepared by replacing Se/TBP with Te/TBP in the same concentration, while the other parameters remained the same.^42^ To reduce the size of Te microstructures, 0.75 mmol of ZnO powder was added to the mixture of IPA and TOPO, while the other parameters remained the same. Synthesis of S nanostructures was achieved by replacing Se/TBP with S/TBP and decreasing the reaction time to 3 min, while other parameters remained the same as Se NRs.

### Characterization

Crystal structures were determined by X-ray diffraction (Shimadzu XRD-6000) using Cu K_α_ radiation. The XRD samples were prepared by dropping Se NRs (or Te microstructures) onto a glass substrate. Scanning electron microscopy (SEM, Hitachi SU8010) images were taken to examine the morphology of the nanomaterials and micromaterials. Transmission electron microscopy (TEM) images were taken using JEOL JEM-ARM200FTH. Energy-dispersive spectroscopy (EDS) attached to SEM or TEM was used to analyze the composition of samples.

## Supplementary Material



## Notes

Photo of the reaction mixture under different conditions; SEM, XRD, and TEM images of Se NRs, Te NRs, and S NRs; TEM-EDS of Te NRs; and band diagrams of the photoconductor on PET (PDF)
