# Supplementary material for: Preparation of Rod- and Urchin-Like Selenium Nanostructures by Hot-Injection and Application to Simple Flexible Coated Photosensors
Source: ACS Omega. 2025 Dec 8;10(50):61762–9. doi: 10.1021/acsomega.5c07956 (PMC12750397; doi:10.1021/acsomega.5c07956)
Supplement: Supplementary file 1 [file ao5c07956_si_001.pdf]

Supporting Information

Preparation of Rod- and Urchin-like Selenium Nanostructures by Hot-injection and Application to Simple Flexible Coated Photosensors

Kapil Patidar[1], Pen-Ru Chen[1], and Hsueh-Shih Chen[1,2]*

[1]National Tsing Hua University, Department of Materials Science and Engineering, No. 101, Sec. 2, Kuan-Fu Road, Hsinchu, 300, TW

[2]College of Semiconductor Research, National Tsing Hua University, No. 101, Sec. 2, Kuan-Fu Road, Hsinchu, 300, TW

[*] Corresponding author: chenhs@mx.nthu.edu.tw

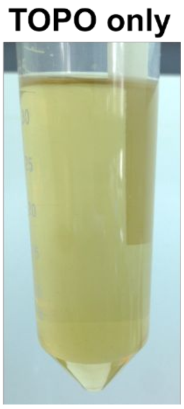

Figure S1. Photo of the final reaction mixture using only TOPO as solvent. The sample color is light-yellow and appears to be transparent without any precipitates.

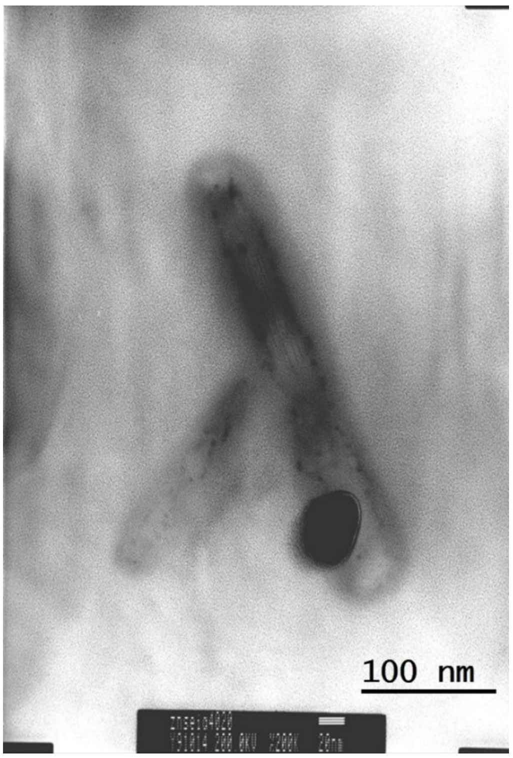

Figure S2. TEM image of a Se NR after exposing to electron beam (back spot in on the Se NR). The NR transforms to a tubular structure.

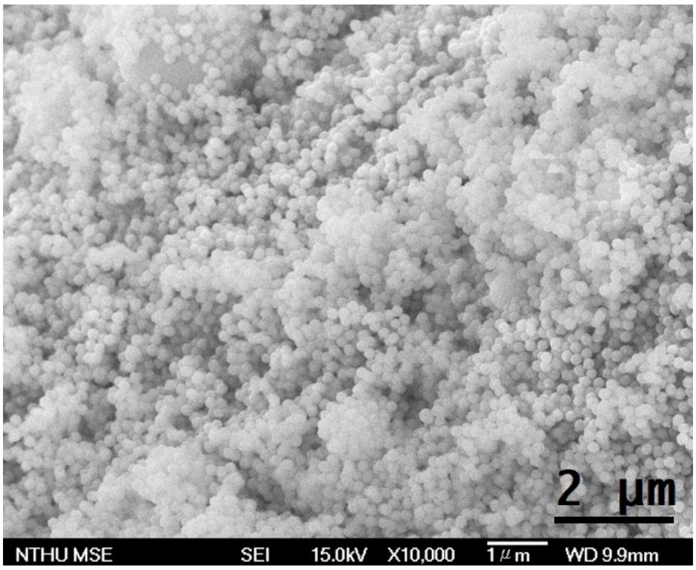

Figure S3. SEM image of Se particles. The particles were confirmed to be Se crystals by XRD.

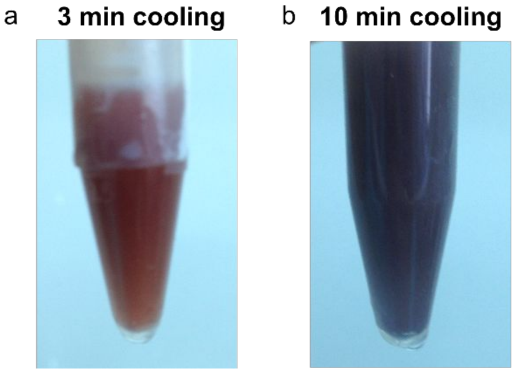

Figure S4. Photographs of the aliquots taken at (a) 3 minutes and (b) 10 minutes during cooling at a slow rate of 30 °C/min. The 3-minute sample exhibits a brick-red color, indicating the formation of amorphous Se (a-Se) particles. In contrast, the 10-minute sample appears dark purple, suggesting the formation of trigonal Se (t-Se)-derived hexagonal Se nanostructures.

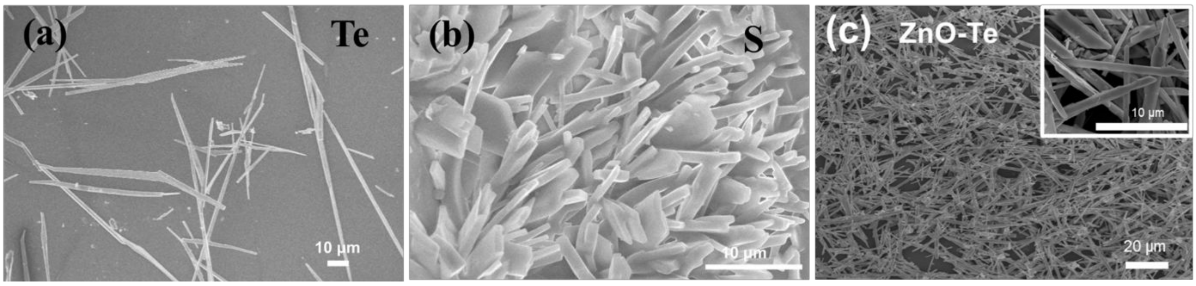

Figure S5. SEM images of (a) Te microrods (Te MRs), (b) sulfur nanorods (S NRs), and (c) ZnO-assisted Te microrods (ZnO-Te MRs) synthesized via the hot-injection cooling process. (c) Adding ZnO yields markedly thinner Te rods: without ZnO, Te MRs show diameters ~5 μm with lengths 60-200 μm; with ZnO, diameters decrease to 0.6-2.5 μm and lengths to 9-16 μm.

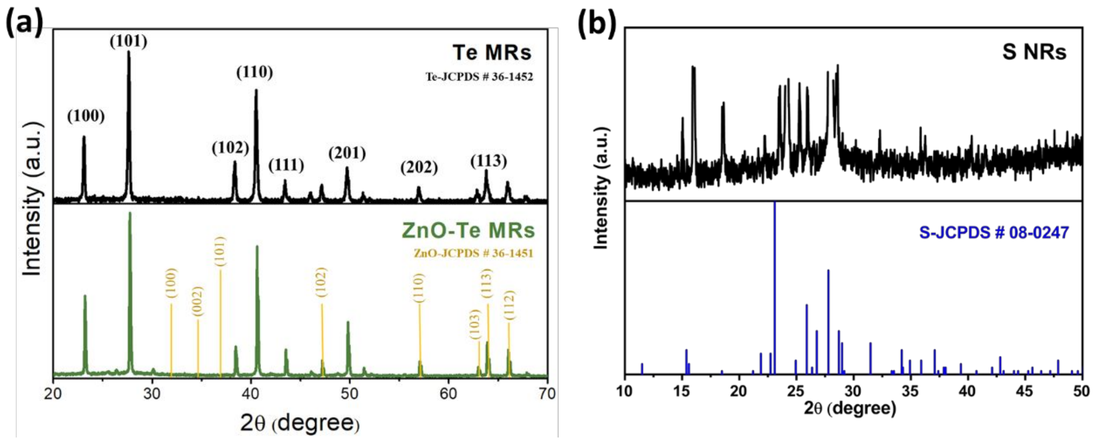

Figure S6. XRD patterns of (a) Te MRs synthesized without and with a ZnO additive (ZnO-Te MRs), and (b) S NRs. In (a), the two traces exhibit essentially identical peak positions and profiles, indicating the same crystalline phase; adding ZnO does not alter Te crystallinity but yields smaller-diameter Te rods.

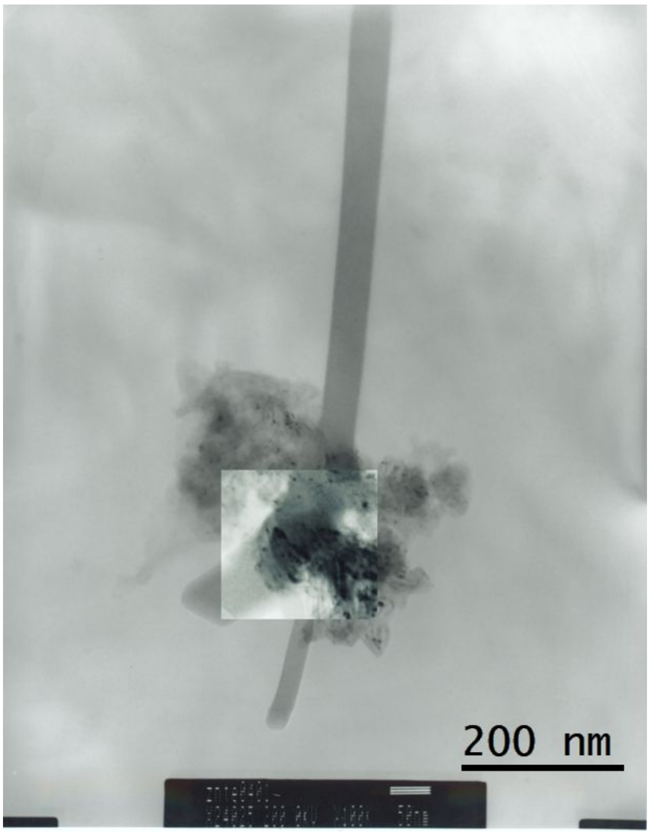

Figure S7. TEM image Te NRs and particles. The square region highlights the connections of NR and particles.

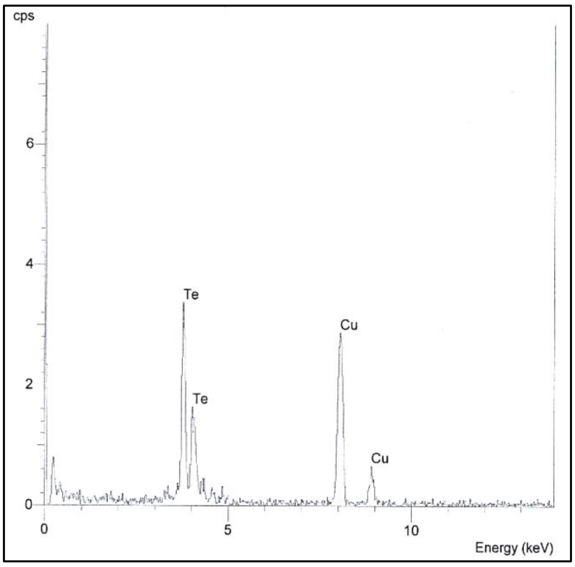

Figure S8. TEM EDS of Te NRs.

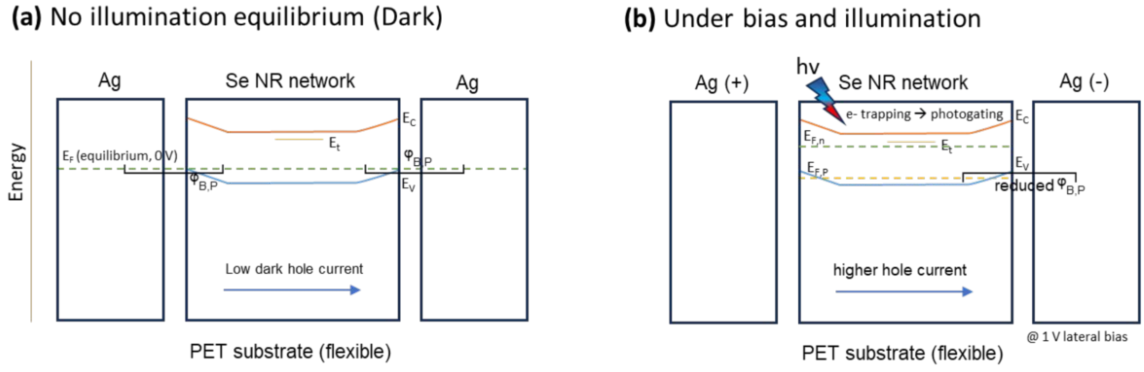

Figure S9. Qualitative band diagrams of the lateral Ag/Se-nanorod network/Ag photoconductor on PET. (a) Dark: p-type t-Se with E_F near E_V; Schottky barriers at Ag/Se and the wide Se bandgap yield a low hole dark current. (b) Under illumination (@ 1V): electron-hole generation with electron trapping (E_t) photogates the channel, lowering the effective hole-injection barrier ($\varphi_{B,P}$) and increasing current (quasi-Fermis $E_{F,n}$ and $E_{F,p}$ are shown in Se).

Note S1:

This diagram illustrates the qualitative operating mechanism of the Ag/Se-NR/Ag photoconductor: hole-dominated transport in p-type t-Se; Schottky-limited injection in the dark; and, under light, trap-assisted photogating that enhances the conductivity of the percolating NR network. Absolute energy positions and barrier heights are not intended to be quantitative. Confining NRs in grooves (main-text Figure 6b) improves percolation/contact and yields higher, more stable photocurrent.
